# Recurrent Plunging Ranula Due to a Sublingual Ectopic Gland: A Rare Clinical Entity

**DOI:** 10.7759/cureus.52590

**Published:** 2024-01-19

**Authors:** Cristina Rodrigues Barros, Frederico Caeiro dos Santos Portugal Guerreiro, Joaquim Seixas-Martins, Maria do Céu Machado

**Affiliations:** 1 Oral Medicine, Centro Hospitalar Universitário de Lisboa Central, Lisbon, PRT; 2 Plastic and Reconstructive Surgery, Centro Hospitalar de Lisboa Ocidental, Lisbon, PRT

**Keywords:** transoral surgical approach, tail sign, plunging ranula, ectopic gland, salivary gland, mouth floor

## Abstract

Ranula is a benign cystic lesion caused by the escape and collection of salivary mucus. Classically, it is divided into simple ranulas, a cystic mass in the floor of the mouth, and diving/plunging/cervical ranulas, a submandibular mass without apparent intraoral involvement. Although plunging ranula is a well-documented cause of neck swelling, its association with the presence of ectopic sublingual glands is extremely rare, with less than five cases reported.

Other cervical cystic lesions may have the same clinical aspect; therefore, advanced diagnostic techniques like a CT scan or MRI play a critical role in early diagnosis. Different approaches have been used to treat ranulas, including non-invasive, minimally invasive, and surgical techniques.

The purpose of this paper is to highlight a case report of a giant plunging ranula due to an anatomical aberration of the right sublingual gland, along with a significant literature review.

## Introduction

The principal etiologies of the main salivary gland’s pathology (parotid, sublingual, and submandibular) are lithiasic and tumoral. Most of the proliferative lesions in the salivary glands are benign, and only less than 20% are malignant [1]. Sublingual glands are the smallest of the major salivary glands. Anatomically, each gland has a row of about 12-20 short ducts that open independently along the summit of the sublingual fold [2].

Among tumoral causes, ranulas are mucoceles (representing about 0.2 cases per 1000 persons) that occur in the floor of the mouth and account for 6% of all oral sialocysts [3-4]. The etiopathogenesis is currently unknown. Common causes that have been described include iatrogenic or secondary trauma and sublingual gland disorders [1]. Congenital origin also represents an uncommon cause, with a prevalence of 0.79% [5].

It is believed that its pathophysiology is explained by mucous escape from the ducts of the Rivinus of the sublingual gland and, infrequently, from the minor salivary glands at the same location [2,6-7]. In addition to this oral floor lesion (simple ranula), another uncommon clinical variant (plunging ranula) exists (only with a few hundred reported cases), resulting from its extension along sublingual space, anteriorly and posteriorly beyond the free edge of/or through the mylohyoid muscle [2,6-7]. Occasionally, it may attain large dimensions due to the involvement of the submandibular and parapharyngeal spaces, which renders it difficult to differentiate from other benign or malignant neck masses. Accurate differentiation through clinical or radiological techniques is critical because some of them may be treated with different approaches [8].

Due to the existing knowledge gap regarding its true etiopathogenesis, plunging ranulas management is a controversial topic, with conflicting evidence as to which treatment modality is best. A variety of different approaches have been quoted in the literature, ranging from aspiration, marsupialization, ranula drainage with sublingual gland excision, and complete or partial excision of the ranula with or without sublingual and submandibular gland excision [9]. The recurrence rate varies according to the procedure performed [10].

We report a case of a giant plunging ranula associated with an ectopic right sublingual gland, successfully treated with a transoral approach, with no record of anatomical or functional surgical complications, as well as recurrence of the initial lesion.

## Case presentation

We describe a 35-year-old male reported to the department of plastic, reconstructive, aesthetic, and maxillofacial surgery with an asymptomatic swelling in the submandibular region, associated with facial aesthetic compromise, which had been present for 12 months. The patient reported an insidious increase in the swelling size. No paresthesia history was reported.

The patient was an ex-smoker (10 pack-years) and had no history of chronic medical illnesses. Family history was not relevant. He reported a lesion and a surgical intervention at the same site five years ago that was treated with the marsupialization of the right sublingual gland.

**Figure 1:**
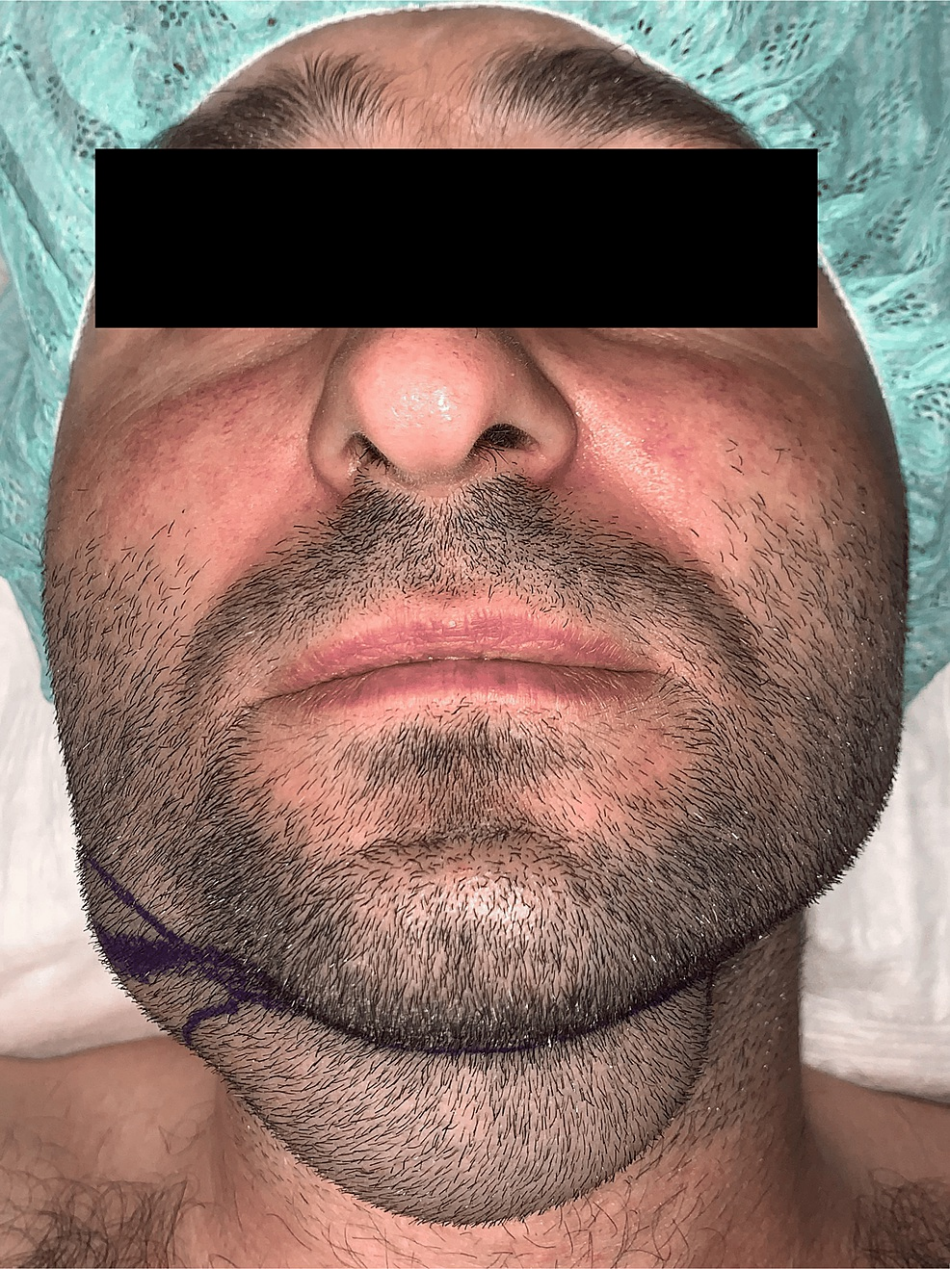
Extraoral view: right and left submandibular swelling

On extraoral examination, the following facial features were observed: facial asymmetry due to a diffuse roughly oval with well-defined margins, swelling in the right submandibular triangle, and in the right and left cervical anterior region (Figure 1).

**Figure 2:**
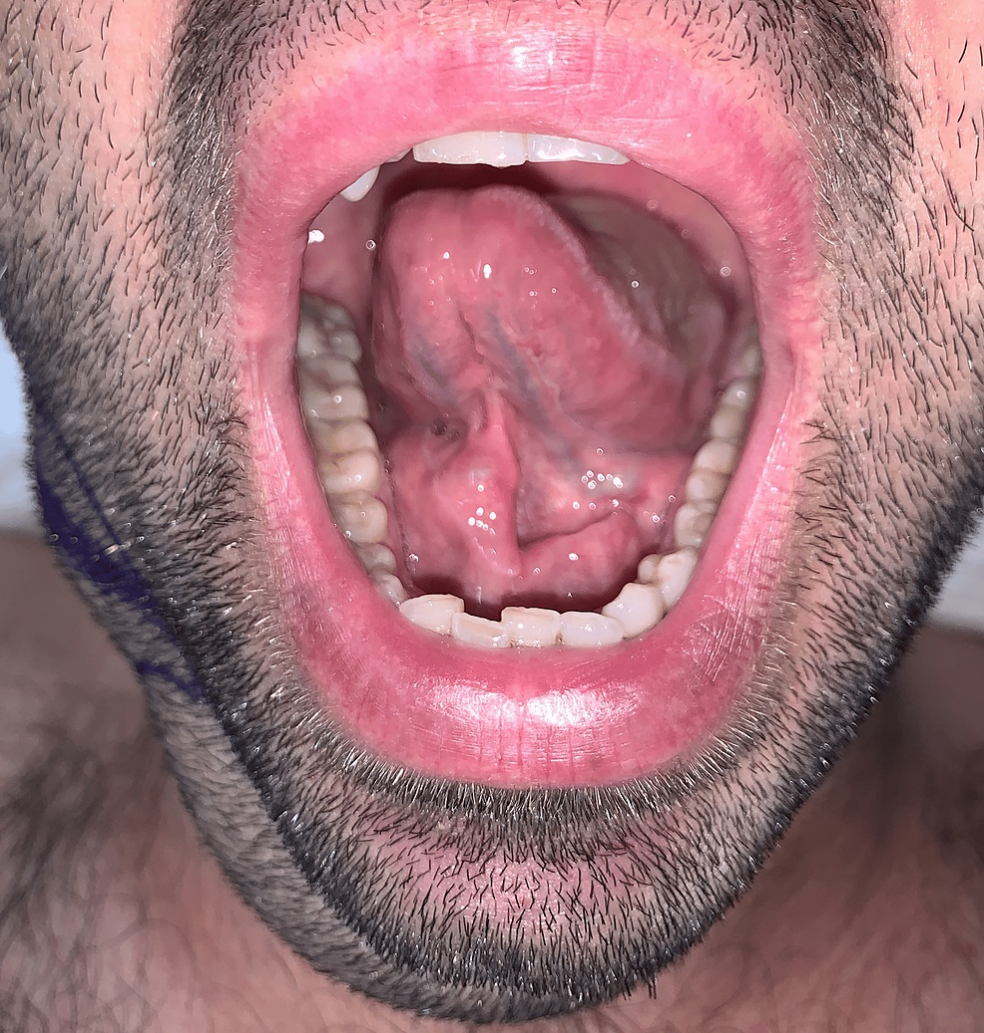
Intraoral view: swelling in one side of the lingual frenum (right floor of the mouth)

On palpation, the swelling was painless, soft, non-tender, and fluctuant. There were no other inflammatory signs. In intraoral examination, there was a smooth and movable mass in the mouth’s floor and lingual frenum’s right side (Figure 2).

No discoloration of the oral mucosa was observed. The salivary right and left ducts were patent. Oral hygiene was adequate. There were no caries lesions or evident periodontal pathology, tooth loss, or a recent history of mandibular implant restoration. Routine blood investigations and thyroid profile (hemoglobin 14.7 g/dL, C-reactive protein 0.1 mg/dL, thyroid-stimulating hormone 3.1 mU/L, free T4 0.9 ng/dL) were normal.

**Figure 3:**
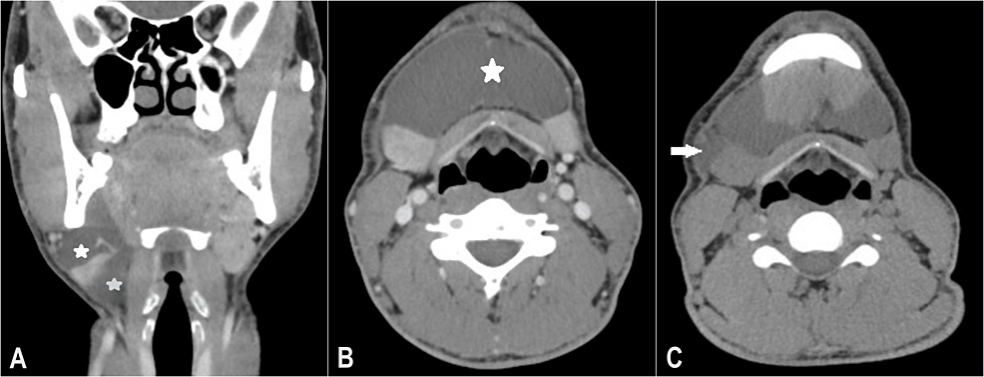
(A) Coronal CT image showing hypodense fluid collection above (white star) and below (blue star) the mylohyoid muscle. (B) Axial CT image with contrast, showing well-defined swelling in the right and left submandibular region (white star). (C) Axial CT image showing “tail sign” phenomenon (white arrow)

**Figure 4:**
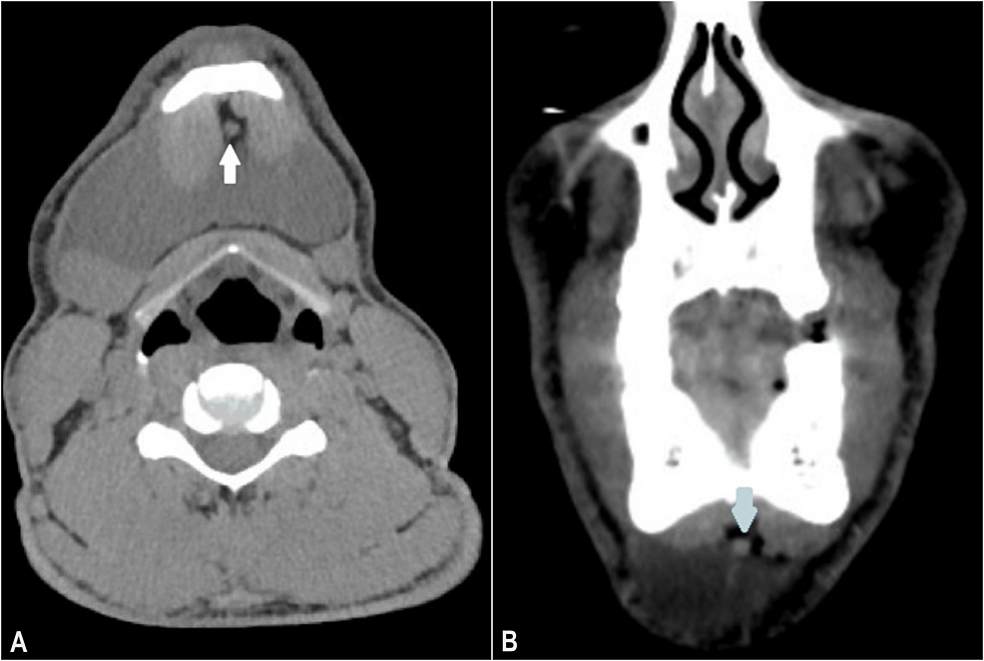
(A) Axial CT image showing the accessory lobe of the right sublingual gland (white arrow). (B) Coronal CT image showing accessory lobe of the right sublingual gland (blue arrow)

Cervical and thoracic CTs were performed with and without water-based iodine contrast. It revealed a 66 x 77 x 40 mm hypodense, homogeneous fluid collection in the right and left submandibular regions, with a deep inferolateral extension through the submental area (Figure 3), close to the right sublingual gland, and a nodular lesion compatible with an accessory lobe of the gland (Figure 4).

The pharyngeal and parapharyngeal spaces were patent, and no pathological ganglionic formations of the cervical, mediastinal, hilar, or axillary chains were reported. Based on the clinical and radiological findings, a provisional diagnosis of plunging ranula was made.

**Figure 5:**
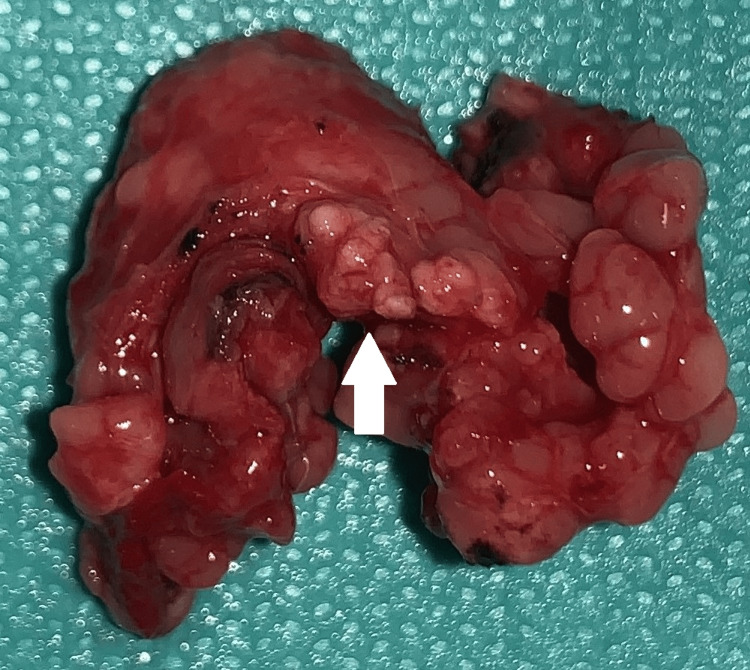
Resected specimen of the right sublingual gland with a white solid nodule (white arrow)

The lesion was addressed under general anesthesia using a transoral technique. Careful precautions were taken not to injure vital structures located on the mouth floor. The whole right sublingual gland was resected (Figure 5), the lingual nerve was secured, and the surgical exploration of the submylohyoid space was followed by spontaneous transoral drainage of the ranula’s content with copious viscous mucin.

Primary closure of the mouth floor was done with the placement of a 24-hour intraoral passive drain. The tissue was subjected to a histopathological study. Macroscopic evaluation revealed a 5.5 x 1.6 x 0.8 cm sublingual gland with a brownish lobulated section. A 0.2 cm white and solid nodule was seen adjacent to the external surface. Microscopic signs revealed moderate chronic inflammatory infiltration, suggestive of chronic sialadenitis, with no signs of malignancy. The nodular tissue was identified as corresponding to an accessory gland lobe.

Acetaminophen 1 g, every eight hours, and metamizol, 575 mg, every 12 hours, were prescribed and carried out for five days. On the day after the surgery, the drain was removed. Transient pain and edema were observed; however, they spontaneously improved within two weeks. A monthly follow-up period was initiated, and three months later, no signs of recurrence were observed. Thereafter, the patient dropped out of follow-up.

## Discussion

The term ranula, derived from the Latin word "rana,” which means “belly of frog,” is used to describe a mucus-filled cavity resulting from either mucous extravasation or, less commonly, a mucous retention cyst derived from the major sublingual or submandibular salivary glands [8,11]. This clinical entity can present at any age, in children and young adults (reported from two to 61 years of age), with a peak frequency in the second decade and a slight female preponderance [4,11].

The etiology of ranula remains unclear. However, some causes have been described: anatomical changes (perforation or dehiscence in the mylohyoid muscle), iatrogenic or secondary trauma (complication of oral surgery involving the mouth floor - sialolith removal, transposition of the Wharton’s duct, dental implant placement), and congenital (presence of an ectopic sublingual gland on the cervical side of the mylohyoid muscle) [1,12].

The pathophysiology is transversal to all causes, explained by hypertension in the excretory duct due to obstruction, leading to an acinar rupture in the salivary gland and then extravasation of the mucus into the surrounding connective tissue. The accumulation of mucous forms a pseudocyst that lacks an epithelial lining [10,13].

According to their location, ranulas have been classically divided into two different clinical categories: simple or plunging. The simple/oral ranula is confined to the mouth floor (sublingual space). In some cases, a variant with moderate incidence appears as a submandibular mass, named diving/plunging/cervical ranula [13]. It occurs when the fluid pressure of the mucin dissects between the mylohyoid and hyoglossus muscles or through congenital dehiscence in the mylohyoid muscle. Furthermore, other areas may be covered, such as submental, cervical, supraclavicular, retropharyngeal, and upper mediastinum spaces [11,14].

The diagnosis of a plunging ranula is usually determined by a combination of history, clinical presentation, and imaging studies [3]. Clinically, it appears as an asymptomatic, continuously enlarging small to medium lesion (4-10 cm) that tends to cause lateral swelling, which can sometimes cross the midline, displacing the tongue, and interfering with oral function. It usually presents as a non-tender, fluctuant mass that may not be well-defined, freely movable, or associated with the thyroid gland or lymph node chains. Although less common, large ranulas or those located in the caruncula sublingualis may lead to partial obstruction of the Wharton’s duct, resulting in submandibular swelling when eating, dysphagia, and airway obstruction [3,13].

Since the diagnosis is not always easy, an assessment with non-invasive or invasive diagnostic methods can be helpful with ultrasonography, CT scans, MRIs, sialograms, and aspiration cytology [10]. CT scans and MRIs are frequently requested. Although MRI is the most sensitive study to evaluate the sublingual gland, an assessment with CT scanning can better outline the precise boundaries of the cyst and its attenuation [1,13]. On a CT scan, the plunging ranula is a unilocular, homogeneous, well-defined, non-enhancing mass with fluid attenuation. Usually, it is located within the submandibular space, with contiguous involvement of the ipsilateral sublingual and parapharyngeal spaces, often ventrally crossing the midline. Communication between the sublingual and submandibular components typically occurs behind the posterior free edge of the mylohyoid muscle. This typically appears as a smooth tapered continuation anteriorly into the sublingual space, the so-called tail sign [1,15-16].

The differential diagnosis of ranula includes a wide variety of clinical entities such as branchial cleft cyst, cystic hygroma, parathyroid cyst, cervical thymic cyst, dermoid cyst, benign teratoma, thyroglossal duct cyst, cystic or neoplastic thyroid disease, submandibular sialadenitis, intramuscular hemangioma, infectious cervical lymphadenopathy (Epstein-Barr virus, cat scratch disease, tuberculosis), hematoma, lipoma, and laryngocele [3,13].

Although many treatments are described for the management of simple ranulas, there is no consensus opinion on the definitive management of the plunging ranula. Multiple options exist, including medical and surgical therapies [17].

The most reported medical approach was sclerotherapy with OK-432 (Picibanil) or bleomycin. It promotes the reduction and/or elimination of the pseudocyst wall in over 90% of cases, with a low frequency of severe complications. Pingyangmycin, another reported conventional sclerosing agent, has been pointed out as an optimal method to treat this lesion [18-19].

Surgery, as the main management option, can be done through a transoral, transcervical, or combined approach. Surgical techniques include simple aspiration, marsupialization, ranula drainage with sublingual gland excision, and complete or partial excision of the ranula, with or without sublingual and submandibular gland excision [9,17].

Simple aspiration, based on transcervical drainage access, followed by submandibular compression, is a technique with good results, although with post-treatment recurrence after around 17 months. Marsupialization can be an alternative, described for smaller superficial cysts. However, high rates of recurrence (61%) are reported [1,19].

Recently, the removal of the secreting tissue that leads to ranula formation has been considered the most efficient treatment [14]. To some authors, performing a sublingual excision is necessary if the lesion measures more than 1.5 cm, as well as a biopsy of the cystic wall for histologic examination. It will allow the exclusion of a squamous cell carcinoma arising from the cystic wall and a papillary cystadenocarcinoma of the sublingual gland, which may present as a ranula too [3,19]. A transcervical approach can lead to a surgical scar, marginal mandibular nerve injury, and Wharton’s duct injury (stenosis and saliva leakage) [14]. For this reason, a transoral technique was considered a preferred alternative with lower risks, except for lingual nerve injury. Transoral complete excision to partial excision of the sublingual gland is preferable, with less risk of the ranula’s recurrence (0% vs. 20%) [19-20]. Another alternative includes sublingual gland excision plus ranula drainage with a drain placement in the sublingual space, which is highlighted as the most effective therapeutic treatment for plunging ranulas in a series of 450 ranulas with a 1% risk of recurrence [9,20].

Up to now, a small number of cases further describe ranula excision along with removal of the sublingual and submandibular glands [9]. Upon review of the reported case, we believe that the chosen surgical approach, ipsilateral sublingual gland excision with ranula evacuation, contributed to the absence of complications. To avoid other major risks due to a more invasive dissection, we considered ranula's excision unnecessary because it was not a true cyst and to guarantee an effective attempt without lingual nerve damage.

## Conclusions

Although the plunging ranulas have been documented with moderate frequency, their diagnosis and differentiation from similar cases with neck swelling are necessary. Along with clinical observation, radiological and histopathological investigations should be carried out promptly to differentiate injuries that require early treatment, such as malignant salivary gland tumors (cystadenocarcinoma) or other specific malignant common lesions of the head and neck region (squamous cell carcinoma).

Treating young patients conservatively through a transoral approach with sublingual excision plus ranula evacuation is a surgical alternative that presents encouraging results, with low recurrences and complication rates and better cosmesis.
